# Peroxynitrite dominates sodium nitroprusside-induced apoptosis in human hepatocellular carcinoma cells

**DOI:** 10.18632/oncotarget.16164

**Published:** 2017-03-13

**Authors:** Ying-Yao Quan, Yu-Hong Liu, Chun-Mei Lin, Xiao-Ping Wang, Tong-Sheng Chen

**Affiliations:** ^1^ Department of Pain Management, The First Affiliated Hospital of Jinan University, Guangzhou, China; ^2^ MOE Key Laboratory of Laser Life Science and College of Life Science, South China Normal University, Guangzhou, China

**Keywords:** apoptosis, HepG2 cells, nitric oxide, peroxynitrite, sodium nitroprusside

## Abstract

This study aims to explore which radicals dominate sodium nitroprusside (SNP)-induced cytotoxicity in human hepatocellular carcinoma (HCC) cells (HepG2 and Hep3B). Exposure of SNP to cell medium produced abundant nitric oxide (NO), superoxide anion (O_2_^·−^), hydrogen peroxide (H_2_O_2_) and iron ions. SNP potently induced caspases activation, mitochondrial membrane permeabilization and apoptosis in HCC cells. In Hep3B cells, pretreatment with NO scavenger (PTIO) did not prevent SNP-induced cytotoxicity. However, in HepG2 cells, SNP-induced cytotoxicity was prevented significantly by pretreatment with PTIO and O_2_^·−^ scavenger, and especially was almost completely blocked by pretreatment with FeTPPS (peroxynitrite scavenger). In contrast, although H_2_O_2_ scavenger potently scavenged SNP-induced H_2_O_2_ production, it did not prevent SNP-induced cytotoxicity in HepG2 cells. In addition, pretreatment with DFO (iron ions chelator) and iron-saturated DFO respectively completely prevented SNP-induced cytotoxicity in HepG2 cells. Collectively, peroxynitrite from the reaction between NO and O_2_^·−^ elicited from SNP dominates the SNP-induced apoptosis of HepG2 cells, in which both iron ions and H_2_O_2_ are not involved.

## INTRODUCTION

Sodium nitroprusside (SNP) is a potent hypotensive agent widely used for treating hypertension-emergencies during surgery and improving heart function after infarction [1]. SNP exposed to light or reducing condition rapidly releases nitric oxide (NO) that [2, 3] can directly relax vascular smooth muscle cells [4, 5]. In addition to the vasorelaxant effect, NO has been demonstrated to be involved in many physiopathological responses, including platelet aggregation, respiration, cell migration, immune response and apoptosis [5–9]. It was reported that overexpression of gene-encoded NO synthase 2/3 (NOS2/3) potently increased the expressions of p53, CD95 and Rho kinase proteins [10, 11]. Excessive NO elicited from NO donor (GSNO, SNAP, or NONOate) increased the expression and activation level of proapoptotic Bax protein [12] and caspases [13, 14], and the loss of mitochondrial membrane potential [14]. Interaction of NO with superoxide anions (O_2_^·−^) produces peroxynitrite (ONOO^−^) [15], a much more toxic oxidant than NO, that directly attacks DNA, triggers lipid peroxidation, dissipates mitochondrial membrane potential, and inactivates the complexes I, II, III and V of respiratory chain [16, 17]. ONOO^−^ can also promote cell death via triggering both caspase-dependent (e.g., cytochrome c, APAF-1, Smac) and caspase-independent (especially apoptosis-inducing factor) apoptotic signal pathways [18].

NO elicited from SNP is generally considered to be the key mediator responsible for the toxicological effects of SNP [19–22]. Based on the findings that SNP induced exogenous NO generation and apoptosis in chondrocytes, Blanco for the first time concluded that NO released from SNP dominated SNP-induced chondrocyte apoptosis [19]. This notion was widely adopted to study the molecular mechanism of SNP-induced apoptosis in various cell lines [20–22]. However, we and other research groups found that reactive oxygen species (ROS), the by-product of SNP independent of NO, mediated SNP-induced cytotoxicity in various kinds of cells [23–29]. Our recent studies firmly demonstrated that hydroxyl radicals (^·^OH) from the Fenton reaction between iron ions and hydrogen peroxide (H_2_O_2_) dominated the SNP-induced NO-independent chondrocytes apoptosis though SNP induced NO production [23]. Based on the potent inhibitory effect of uric acid, an ONOO^−^ scavenger, on the cytotoxicity of SNP in mouse macrophage-like RAW264.7 cells some researchers speculated that ONOO^−^ might play a key role in SNP-induced cytotoxicity [30, 31].

This study is designed to explore what radicals dominate SNP-induced apoptosis in human hepatocellular carcinoma (HepG2 and Hep3B) cells. In Hep3B cells, pretreatment with NO scavenger (PTIO) did not prevented SNP-induced cytotoxicity. However, pretreatment with PTIO (NO scavenger), SOD (O_2_^·−^ scavenger) and FeTPPS (ONOO^−^ scavenger) respectively markedly prevented SNP-induced cytotoxicity and generations of NO and O_2_^·−^ as well as ONOO^−^. Although CAT (H_2_O_2_ scavenger) significantly prevented SNP-induced hydrogen peroxide (H_2_O_2_), it did not prevent SNP-induced cytotoxicity. Moreover, both DFO (iron ions chelator) and iron-saturated DFO exhibited superior inhibitory effects on SNP-induced ONOO^−^ generation and apoptosis over PTIO and SOD. These findings demonstrate that ONOO^−^ from the reaction between NO and O_2_^·−^ dominates SNP-induced apoptosis independent of both iron ions and H_2_O_2_in HepG2 cells.

## RESULTS

### SNP induces cytotoxicity of HCC cells

**Figure 1:**
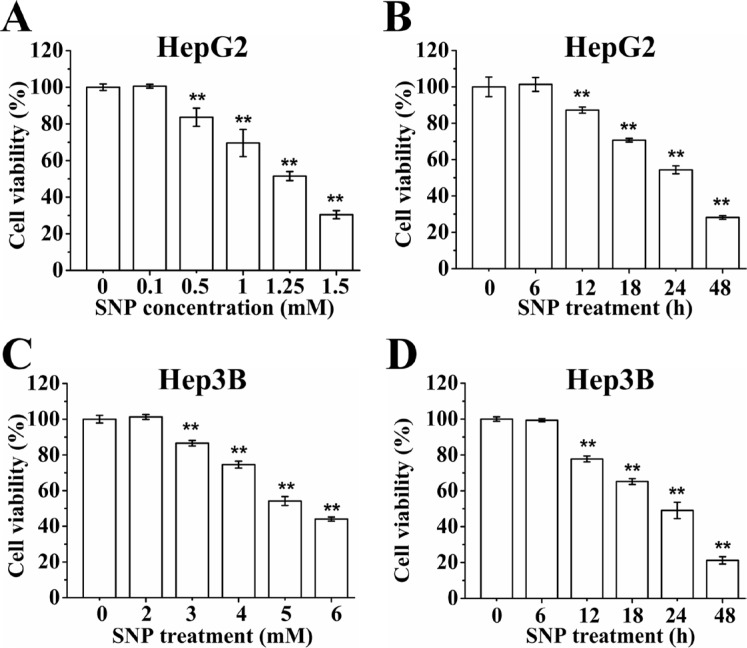
SNP induces cytotoxicity of HepG2 and Hep3B cells (**A** and **B**) SNP induced dose- (A) and time-dependent (B) cytotoxicity of HepG2 cells. (**C** and **D**) SNP induced dose- (C) and time-dependent (D) cytotoxicity of Hep3B cells. Those results represent duplicates with three independent experiments. ^**^P < 0.01 vs Control.

CCK-8 assay showed that exposure of HepG2 cells to different concentration (0–1.5 mM) of SNP for 24 h induced a dose-dependent cytotoxicity (Figure 1A), and exposure of cells to 1.25 mM of SNP for different times (0–48 h) induced a time-dependent cytotoxicity (Figure 1B). 1.25 mM SNP was adopted in following experiments without indication in HepG2 cells. Similar experiments in Hep3B cells also showed that SNP induced dose-dependent cytotoxicity (Figure 1C) and time-dependent cytotoxicity (Figure 1D). 5 mM SNP was adopted in following experiments without indication in Hep3B cells.

### NO mediates SNP-induced cytotoxicity in HepG2 cells

**Figure 2:**
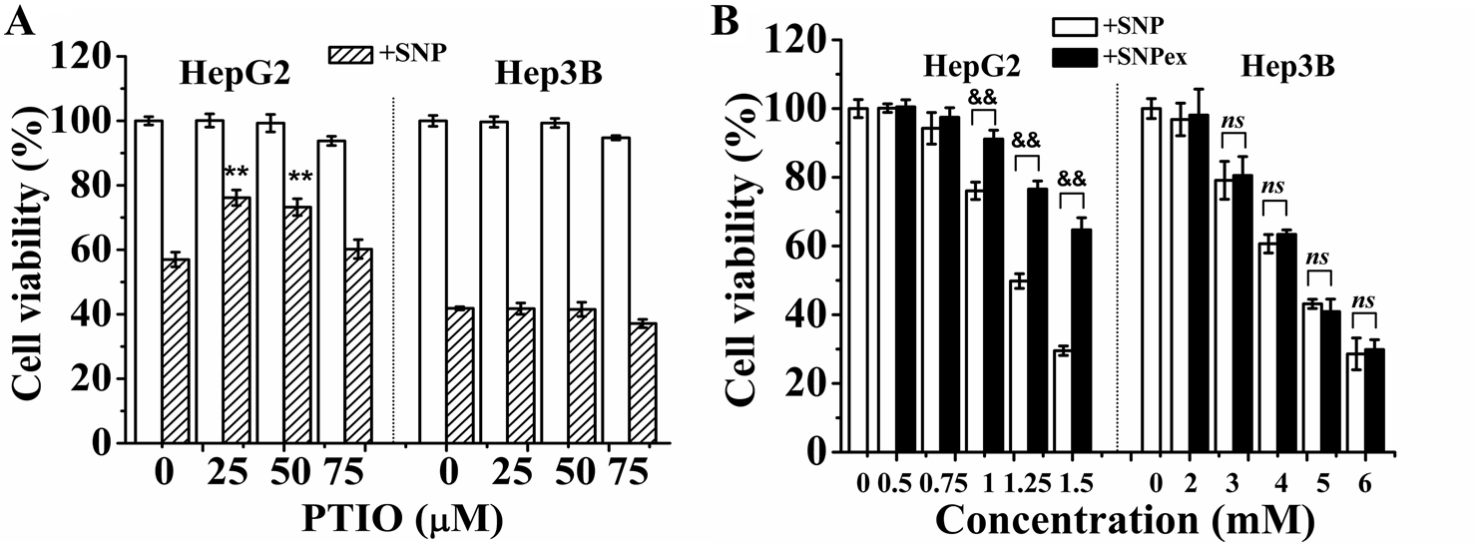
NO mediates SNP-induced cytotoxicity in HepG2 cells (**A**) PTIO pretreatment inhibited SNP-induced cytotoxicity in HepG2 cells but not Hep3B cells. (**B**) SNP induced much more cytotoxicity than SNPex in HepG2 cells, and SNPex induced similar cytotoxicity as SNP in Hep3B cells. Those results represent duplicates with three independent experiments. *ns*: no statistical significance. ^**^P < 0.01 vs Control. ^&&^*P* < 0.01.

Exposure of SNP to cell medium containing fetal bovine serum for 1, 8, 16 and 24 h respectively induced a time-dependent increase of the nitrite/nitrate content (Supplementary Figure 1A). We also used FCM analysis with DAF-FM DA staining to detect the intracellular NO level, and found that SNP treatment for 1 h induced a 36 ± 6.2 % of increase in intracellular NO level that reached to peak at 8 h after SNP treatment (Supplementary Figure 1B). Pretreatment with 25 μM of PTIO, a NO scavenger, completely prevented the SNP-induced NO production (Supplementary Figure 1C) and potently inhibited SNP-induced cytotoxicity in HepG2 cells (Figure 2A), demonstrating the important role of NO in SNP-induced cytotoxicity in this cell line. In contrast, PTIO pretreatment did not prevent SNP-induced cytotoxicity in Hep3B cells (Figure 2A), demonstrating that NO was not participate in SNP-induced cytotoxicity of Hep3B cells. Our previous study has indicated that 24-h-photodegreaded SNP (SNPex) released NO moiety completely [23]. We here found that SNP induced much more cytotoxicity (Figure 2B) than SNPex in HepG2 cells, further confirming the key role of NO in SNP-induced apoptosis in this cell line. However, in Hep3B cells, SNPex induced the same cytotoxicity as SNP (Figure 2B), further demonstrating that NO did not participate in SNP-induced cytotoxicity in Hep3B cells. Collectively, NO mediates SNP-induced cytotoxicity in HepG2 cells.

Further experiments in Hep3B cells show that ROS instead of NO play a dominant role in SNP-induced apoptosis in this cell line (private data), which is similar to our recent report in chondrocytes [23]. Therefore, we here focus on exploring how SNP induces apoptosis in HepG2 cells.

### NO mediates SNP-induced apoptosis

**Figure 3:**
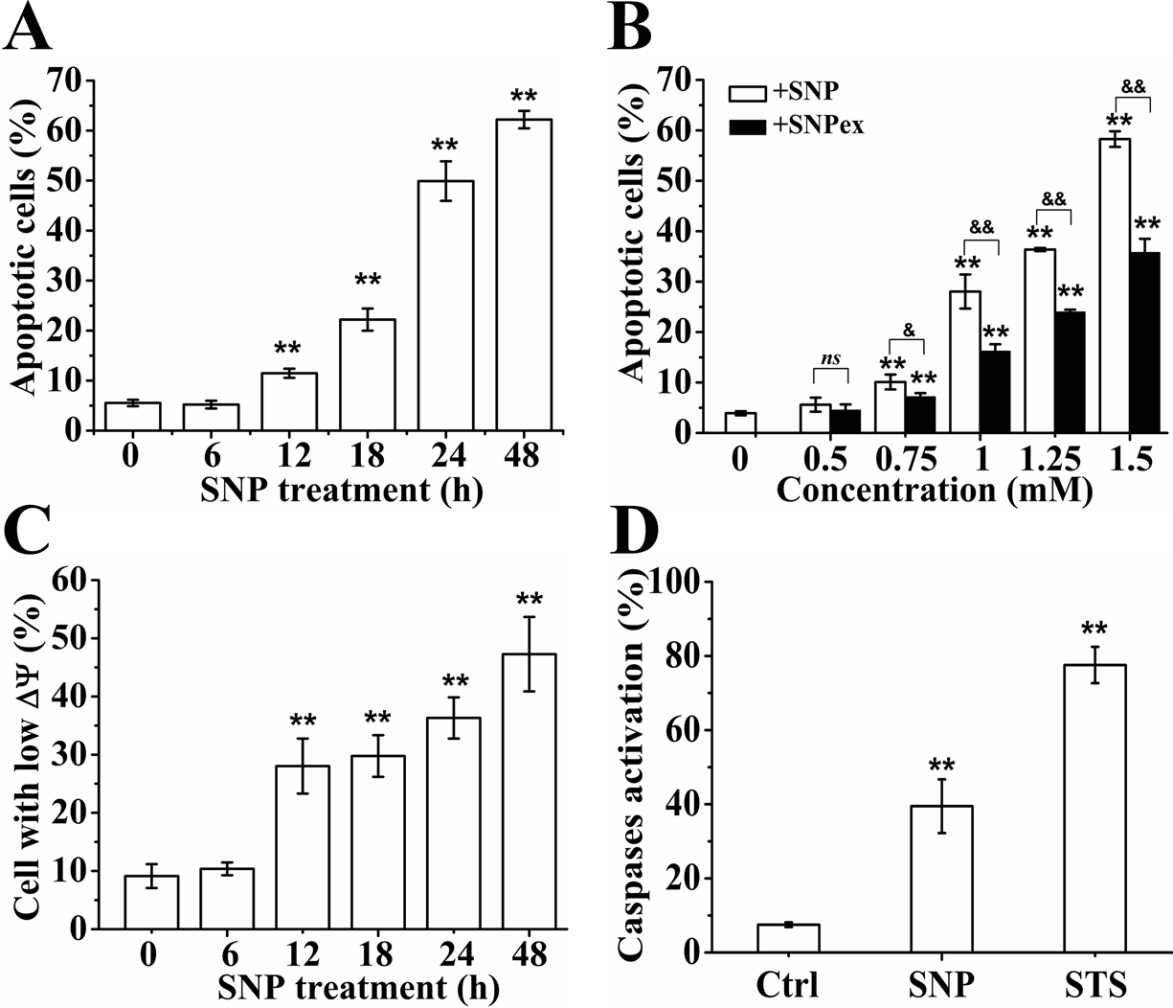
NO mediates SNP-induced apoptosis (**A**) SNP induced time-dependent apoptosis in HepG2 cells. (**B**) SNP induced much more apoptosis than SNPex. (**C**) SNP induced time-dependent loss of ΔΨm. (**D**) SNP induced remarkable caspases activation. Those results represent duplicates with three independent experiments. *ns*: no statistical significance. ^**^P < 0.01 vs Control. ^&&^*P* < 0.01.

Flow cytometry (FCM) analysis with Annexin V-FITC/PI staining was used to assess the form of cell death induced by SNP. Supplementary Figure 2A shows a representative dot-plot showing a time-dependent increase in apoptotic cells (Q2 (PI positive and Annexin V-FITC positive) + Q4 (PI negative and Annexin V-FITC positive)) from 5.5 % (control) to 9.9 %, 19.7 %, 49.8 % and 60.2 % after SNP treatment for 12, 18, 24 and 48 h, respectively, and statistical results from three independent experiments are shown in Figure 3A. In accordance with CCK-8 assay (Figure 2B), we here found that SNP induced much more apoptosis than SNPex (Figure 3B), further confirming the key role of NO in SNP-induced apoptosis in HepG2 cells. In addition, FCM analysis with Rho 123 staining showed that SNP induced a time-dependent decrease of mitochondrial membrane potential (ΔΨm) (Supplementary Figure 2B and Figure 3C), indicating the permeabilization of mitochondrial outer membrane. FCM analysis with FITC-VAD-FMK staining showed a significant increase of cells with activated caspases from 7.6 % (Control) to 43.6 % after SNP treatment (Supplementary Figure 2C), and statistical results from three independent experiments showed that SNP induced a remarkable caspases activation (Figure 3D), demonstrating that caspases were involved in SNP-induced apoptosis. Collectively, NO mediates SNP-induced apoptosis through caspase-dependent mitochondrial apoptosis pathway of HepG2 cells.

### O_2_^·−^ instead of H_2_O_2_ mediates SNP-induced cytotoxicity of HepG2 cells

**Figure 4:**
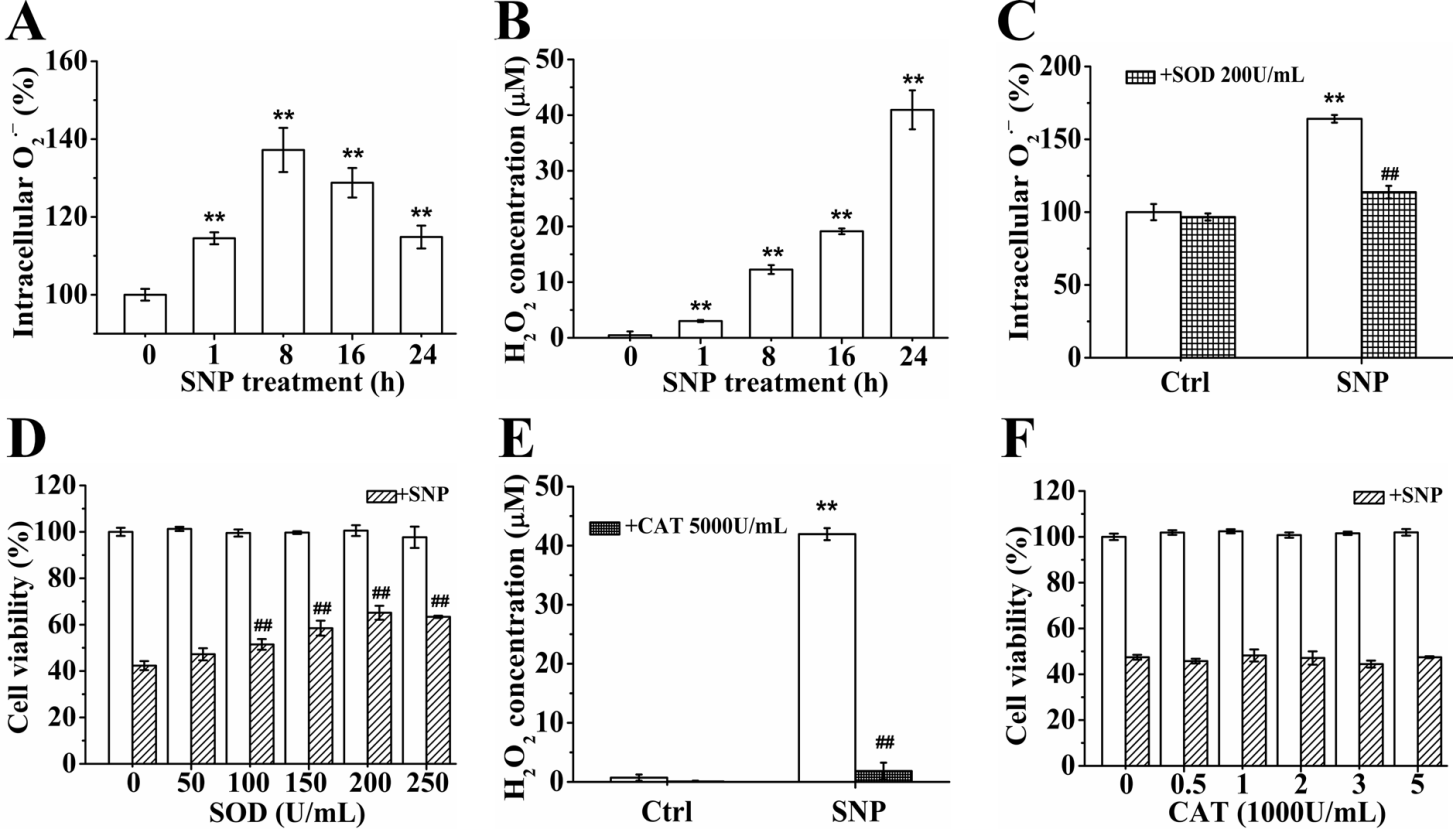
O_2_^·−^ instead of H_2_O_2_ plays an important role in SNP-induced cytotoxicity (**A**) SNP induced intracellular O_2_^·−^ generation. (**B**) SNP induced time-dependent H2O2 generation. (**C**) SOD pretreatment completely inhibited SNP-induced O_2_^·−^ generation. (**D**) SOD pretreatment significantly prevented SNP-induced cytotoxicity. (**E**) CAT pretreatment completely inhibited SNP induced H_2_O_2_ generation. (**F**) CAT pretreatment did not prevent SNP-induced cytotoxicity. Those results represent duplicates with three independent experiments. ^**^P < 0.01 vs Control. ^##^*P* < 0.01vs SNP.

FCM analysis with DHE staining showed that SNP treatment for 1 h resulted in a significant increase of intracellular O_2_^·−^, and the O_2_^·−^ level reached to a peak at 8 h after SNP treatment (Figure 4A). SNP also induced a time-dependent increase in H_2_O_2_ level, and the concentration of H_2_O_2_ in cell medium increased from 0.5 ± 0.7 μM/L (control) to 41.0±3.5 μM/L after SNP treatment for 24 h (Figure 4B). FCM analysis showed that pretreatment with 200 U/mL SOD (O_2_^·−^ scavenger) completely prevented the SNP-induced O_2_^·−^ production (Figure 4C), and also potently prevented SNP-induced cytotoxicity (Figure 4D), indicating the important role of O_2_^·−^ in the SNP-induced cytotoxicity. In contrast, pretreatment with 5000 U/mL CAT (an H_2_O_2_ scavenger) completely prevented SNP-induced H_2_O_2_ production (Figure 4E), but did not show any inhibitory effect on SNP-induced cytotoxicity (Figure 4F), illustrating that H_2_O_2_ was not involved in SNP-induced cytotoxicity. These data demonstrated that O_2_^·−^ instead of H_2_O_2_ played an important role in SNP-induced cytotoxicity.

### ONOO^−^ dominates SNP-induced apoptosis

**Figure 5:**
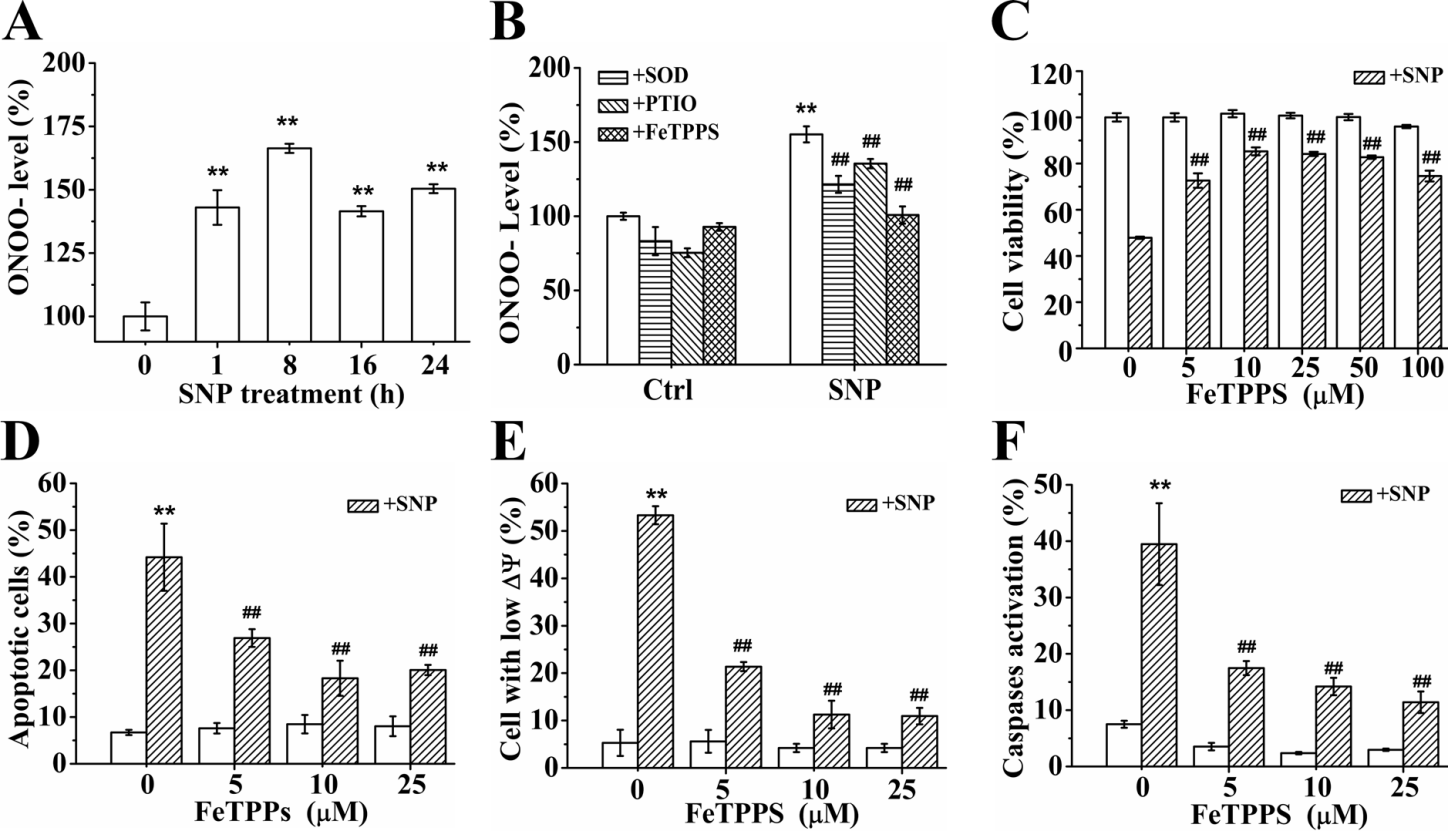
ONOO^−^ dominates SNP-induced apoptosis (**A**) SNP induced intracellular ONOO^−^ generation. (**B**) Inhibitory effects of pretreatment with SOD, PTIO and FeTPPS respectively on SNP-induced ONOO^−^ generation. (**C**–**F**) FeTPPS pretreatment potently inhibited SNP-induced cytotoxicity (C), apoptosis (D), decrease in ΔΨm (E) and caspases activation (F). Those results represent duplicates with three independent experiments. ^**^P < 0.01 vs Control. ^##^*P* < 0.01vs SNP.

It is well known that NO can react with O_2_^·−^ to produce the highly reactive nitrogen species ONOO^−^ [15]. As expected, FCM analysis with DHR 123 staining showed that SNP treatment for 1 h potently induced abundant ONOO^−^ generation (Figure 5A). In order to evaluate the effect of ONOO^−^ on SNP- induced apoptosis, we assessed the effect of FeTPPS, an ONOO^−^ decomposition catalysts (ONOO^−^ specific scavengers), on the cytotoxicity of SNP. Pretreatment with PTIO (NO scavenger) and SOD (O_2_^·−^ scavenger) respectively significantly prevented SNP-induced ONOO^−^ generation, indicating that the reaction of NO and O_2_^·−^ elicited from SNP produced ONOO^−^. Pretreatment with FeTPPS completely prevented SNP-induced ONOO^−^ generation (Figure 5B). CCK-8 assay showed that pretreatment with 10 μM FeTPPS potently antagonized SNP-induced cytotoxicity (Figure 5C), illustrating the key role of ONOO^−^ in SNP-induced cytotoxicity. Similarly, FeTPPS pretreatment also potently antagonized SNP-induced apoptosis (Figure 5D), loss of ΔΨm (Figure 5E) and caspases activation (Figure 5F), further confirming the dominant role of ONOO^−^ in SNP-induced apoptosis of HepG2 cells.

### ONOO^−^ has more cytotoxicity than NO

**Figure 6:**
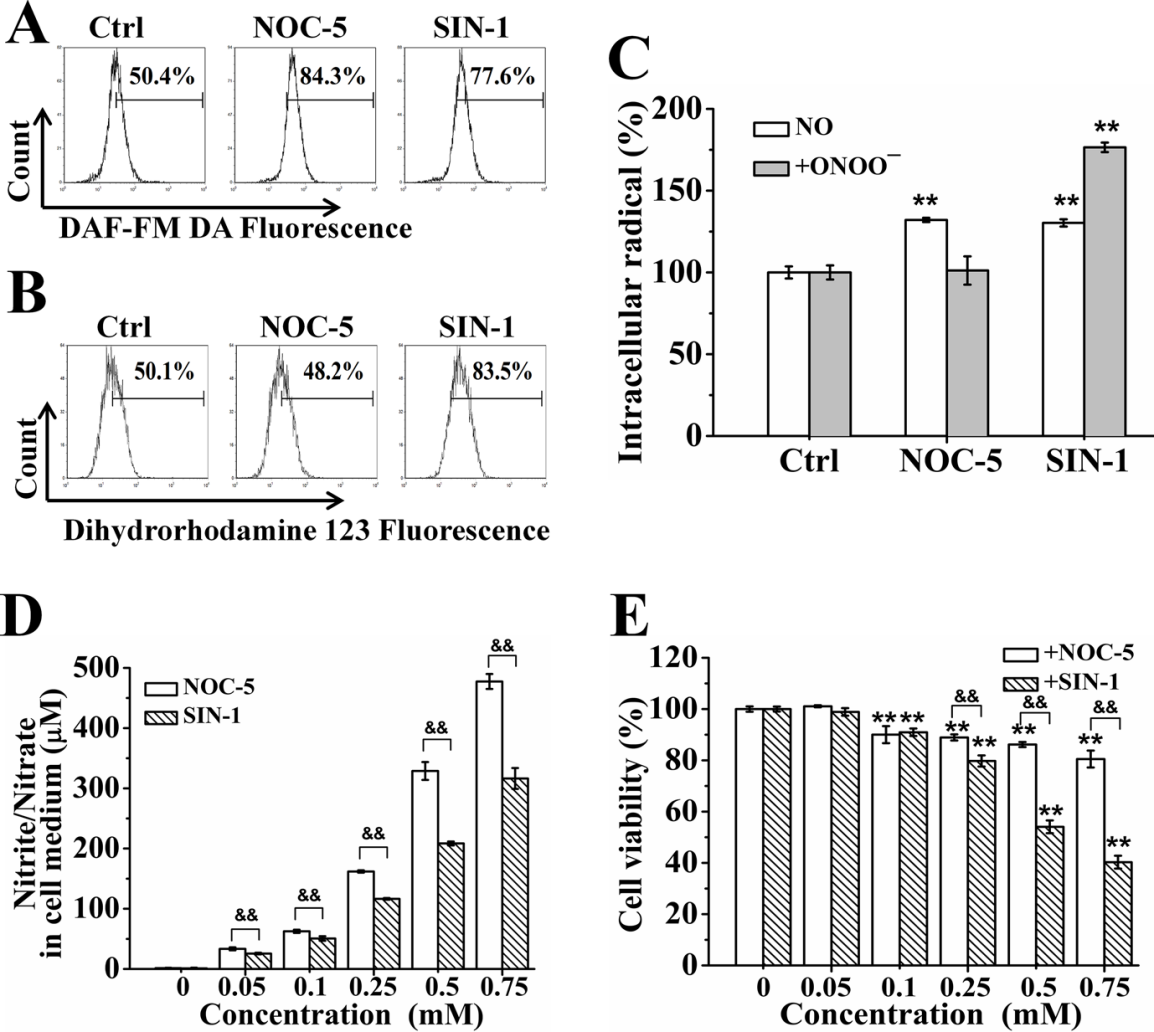
ONOO^−^ has more cytotoxicity than NO (**A**) Both NOC-5 and SIN-1 induced NO generation. (**B**) SIN-1 but not NOC induced ONOO^−^ generation. (**C**) Statistical results on the relative increase of both NO and ONOO^−^ production induced by NOC-5 and SIN-1, respectively. (**D**) NOC-5 induced more nitrite/nitrate generation than SIN-1. (**E**) SIN-1 induced much more cytotoxicity than NOC-5. Those results represent duplicates with three independent experiments. NS: no statistical significance. ^**^P < 0.01 vs Control. ^&&^*P* < 0.01.

To further determine the role of NO and ONOO^−^ in SNP-induced cytotoxicity, we assessed the cytotoxicity of both NOC-5 and SIN-1, two NO donors. It was reported that NOC-5 only released NO during its decomposition, while SIN-1 released both NO and O_2_^·−^, and thus for the continuous formation of ONOO^−^ [32, 33]. FCM analysis showed that both NOC-5 and SIN-1 potently induced NO production (Figure 6A), SIN-1 also potently induced ONOO^−^production whereas NOC-5 did not induce ONOO^−^ generation (Figure 6B). Figure 6C shows the statistical results from three independent experiments on the relative increase of both NO and ONOO^−^ productions induced by NOC-5 and SIN-1, respectively. Although SIN-1 induced less NO production than NOC-5 (Figure 6D), SIN-1 had much more cytotoxicity than NOC-5 (Figure 6E), further supporting the notion that HepG2 cells were more sensitive to ONOO^−^ than NO.

### Iron ions are not involved in SNP-induced cytotoxicity

**Figure 7:**
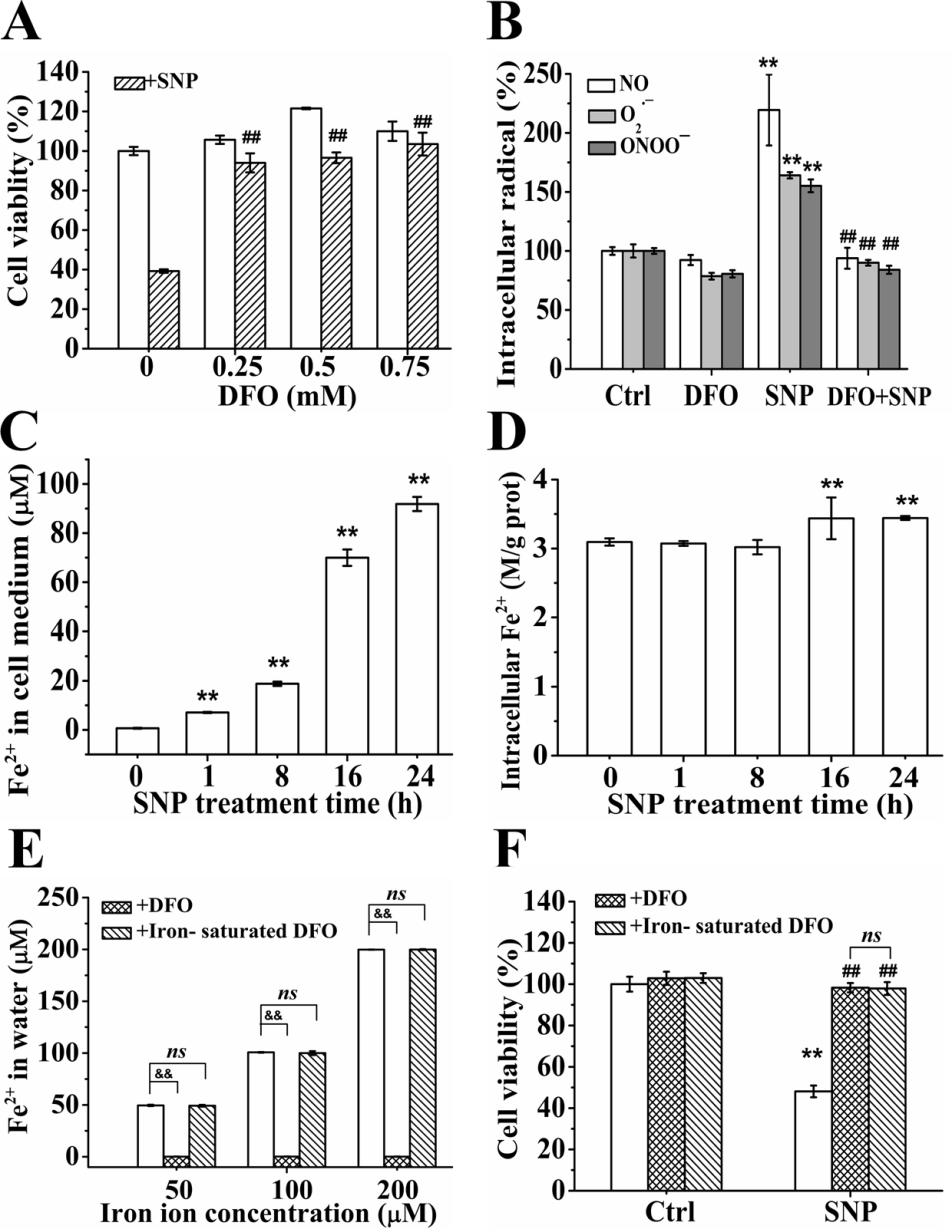
Iron ions are not involved in SNP-induced cytotoxicity (**A**) DFO completely antagonized SNP-induced cytotoxicity. (**B**) DFO completely prevented SNP-induced NO, O_2_^·−^ and ONOO^−^ generations. (**C**) SNP induced time-dependent extracellular Fe2+ generation. (**D**) SNP treatment for more than 16 h induced statistically significant intracellular Fe^2+^ generation. (**E**) Iron ions chelating effect of DFO and iron-saturated DFO. (**F**) Both DFO and Iron-saturated DFO completely antagonized SNP-induced cytotoxicity. Those results represent duplicates with three independent experiments. NS: no statistical significance. ^**^P < 0.01 vs Control. ^##^*P* < 0.01 vs SNP. ^&&^*P* < 0.01.

We firstly assessed the effect of DFO, an iron ion chelator, on the cytotoxicity of SNP, and found that DFO larger than 0.25 mM almost completely prevented the SNP-induced cytotoxicity (Figure 7A). We next assessed the effects of 0.75 mM DFO on SNP-induced generation of radicals including NO, O_2_^·−^ and ONOO^−^, and found that DFO completely inhibited SNP-induced NO, O_2_^·−^ and ONOO^−^ generations (Figure 7B). These results demonstrated that DFO prevented SNP-induced cytotoxicity via scavenging NO, O_2_^·−^ and ONOO^−^.

SNP induced a time-dependent extracellular Fe^2+^ generation in cell medium (Figure 7C), and also significantly increased intracellular Fe^2+^ level after treatment for 16 h (Figure 7D). DFO completely chelated iron ions (50–200 μM) in water, while iron-saturated DFO did not show any iron ions chelating effect (Figure 7E). However, iron-saturated DFO almost completely prevented SNP-induced cytotoxicity (Figure 7F), indicating that iron ions did not participate in SNP-induced cytotoxicity.

### NAC promotes the cytotoxicity of SNP

**Figure 8:**
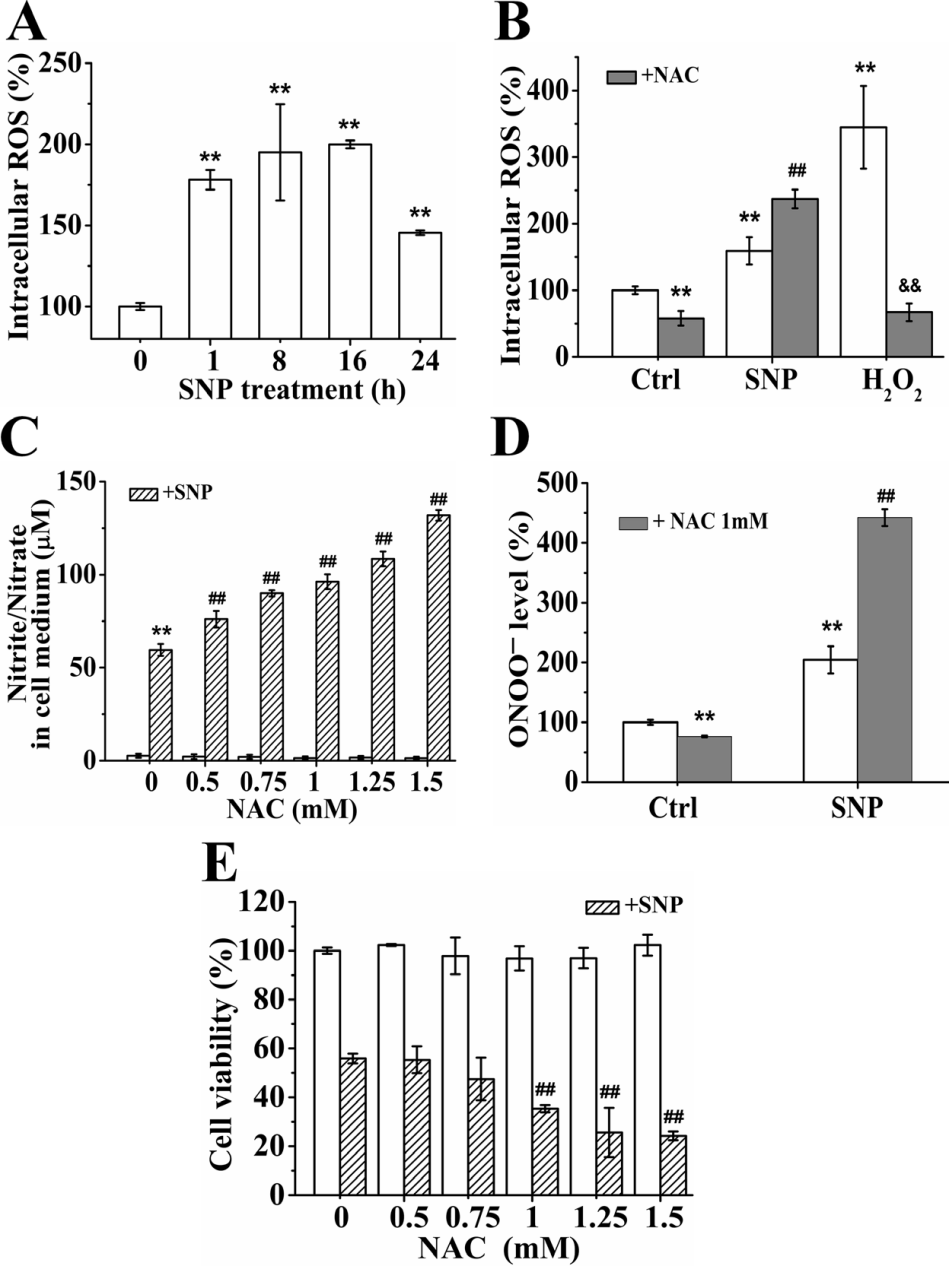
NAC promotes the cytotoxicity of SNP (**A**) SNP induced intracellular ROS generation. (**B**) NAC pretreatment enhanced SNP-induced ROS generation. (**C**) NAC promoted SNP-induced nitrite/nitrate generation in dose-dependent manner. (**D**) NAC potently enhanced SNP-induced intracellular ONOO^−^ generation. (**E**) NAC enhanced SNP-induced cytotoxicity. Those results represent duplicates with three independent experiments. ^**^P < 0.01 vs Control. ^##^*P* < 0.01 vs SNP. ^$$^*P* < 0.01 vs H_2_O_2_.

FCM analysis with DCFH-DA, a ROS probe, showed that SNP also potently induced ROS generation (Figure 8A). Although pretreatment with 1 mM of NAC, a widely used ROS scavenger [24, 28], for 2 h could completely scavenged 500 μM of H_2_O_2_, it did significantly enhance SNP-induced ROS generation (Figure 8B). Moreover, NAC pretreatment significantly enhanced SNP-induced nitrite/nitrate (Figure 8C) and ONOO^−^ generations (Figure 8D). CCK-8 assay showed that pretreatment with NAC more than 1 mM significantly enhanced the cytotoxicity of SNP. These data illustrated that NAC promoted the cytotoxicity of SNP by enhancing the SNP-induced ONOO^−^ level.

## DISCUSSION

This report for the first time demonstrates that ONOO^−^ dominates SNP-induced apoptosis in HepG2 cells. Exposure of SNP to cell medium containing fetal bovine serum produced abundant NO, O_2_^·−^, iron ions and H_2_O_2_. Reaction of NO and O_2_^·−^ formed ONOO^−^ with superior cytotoxicity over NO and O_2_^·−^ towards HepG2 cells. However, both iron ions and H_2_O_2_ did not participate in the SNP-induced cytotoxicity in this cell line. Interestingly, DFO exhibited a very excellent protective effect on the cytotoxicity of SNP via scavenging NO, O_2_^·−^, and ONOO^−^.

Our observations that SNP induces abundant ONOO^−^ production (Figure 5A) and FeTPPS almost completely antagonizes SNP-induced cytotoxicity (Figure 5C) illustrate the dominant role of ONOO^−^ in SNP-induced apoptosis of HepG2 cells. NO has been considered to be a key factor to mediate SNP-induced cytotoxicity [19–22], which was further verified by our findings that PTIO obviously inhibited SNP-induced NO production and cytotoxicity (Supplementary Figure 1C and 2A) as well as SNPex induced less cytotoxicity than SNP (Figures 2B and 3B). However, many studies have demonstrated that the direct toxicity of NO is very modest [27, 33]. Our recent study also showed that although SNP induced NO generation, ROS instead of NO mediated SNP-induced chondrocytes apoptosis [23]. Moreover, NOC-5 which induced more 50-fold than the SNP-induced NO production exhibited much lower cytotoxicity than that of SNP in chondrocytes [23]. Therefore, it is speculated that many potentially toxic effects of NO are more likely to be mediated by its oxidation products rather than NO itself [18].

Although O_2_^·−^, the product of a one-electron reduction of oxygen, is generally poorly reactive and can only attack a few molecules [34], the fact that SOD pretreatment obviously inhibits SNP-induced cytotoxicity (Figure 4C) demonstrates the important role of O_2_^·−^ in SNP-induced cytotoxicity in HepG2 cells. NO can react with O_2_^·−^ to produce ONOO^−^ with a reaction rate larger than 6.7 × 10^9^ M^−l^·s^−l^, so that nearly every collision between O_2_^·−^ and NO results in the irreversible formation of ONOO^−^ [35, 36], and a 10-fold increase in O_2_^·−^ and NO will increase 100-fold of ONOO^−^ formation [18, 37]. Based on the facts that SNP induces NO, O_2_^·−^ and ONOO^−^ generation (Supplementary Figure 1A, Figures 4A and 5A) and PTIO/SOD modestly but FeTPPS almost completely inhibit SNP-induced ONOO^−^ generation (Figure 5B) and cytotoxicity (Figures 2A, 4D and 5C), it is reasonable to consider that SNP firstly releases NO and O_2_^·−^, and then rapidly forms ONOO^−^ to dominate SNP-induced apoptosis in HepG2 cells.

Moreover, although SOD or PTIO pretreatment completely prevented the SNP-induced O_2_^·−^ or NO production (Figure 4C and Supplementary Figure 1A), either PTIO or SOD does not completely antagonized SNP-induced cytotoxicity (Figures 2A and 4D) and ONOO^−^ generation (Figure 5B). This may be ascribed to the different reaction rates between SOD/PTIO and O_2_^·−^/NO as well as between O_2_^·−^ and NO. Consistent with our results, Goldstein and colleagues reported that the formation of ONOO^−^ could not be efficiently inhibited by PTIO even under relatively low fluxes of NO and O_2_^·−^ and millimolar levels of PTIO [38]. Beckman and colleagues reported that the reaction rate of O_2_^·−^ with SOD to produce H_2_O_2_ was ~2 × 10^9^ M^−l^·s^−l^ [35], lower than the reaction rate of NO and O_2_^·−^ to produce ONOO^−^ [35, 36], which may be the reason why SOD could only partially scavenge SNP-induced ONOO^−^ (Figure 5B). Furthermore, ONOO^−^ is such a potent and versatile oxidant that can attack a wide range of biological targets [16] and induce apoptosis in various kinds of cells [39, 40]. Based on the fact that FeTPPS completely scavenged SNP-induced ONOO^−^ generation (Figure 5B), it was reasonable that FeTPPS pretreatment almost completely antagonized SNP-induced cytotoxicity (Figure 5C–5F), exhibiting superior inhibitory effects on SNP-induced cytotoxicity over PTIO and SOD (Figures 5C, 2A and 4D).

In contrast to our recent findings that ^·^OH from the Fenton reaction between iron ions and H_2_O_2_ released from SNP played a dominant role in SNP-induced chondrocytes apoptosis [23], we here found that both iron ions and H_2_O_2_ did not participate in the SNP-induced apoptosis in HepG2 cells (Figures 4F and 7F), though SNP did also induce H_2_O_2_ (Figure 4B) and iron ions generations (Figure 7C and 7D). 5000U/ml CAT pretreatment completely prevented SNP-induced H_2_O_2_ production (Figure 4E), but did not show any inhibitory effect on SNP-induced cytotoxicity (Figure 4F). Similarly, iron-saturated DFO without any iron ions chelating effect (Figure 7E) also completely prevented SNP-induced cytotoxicity (Figure 7F). Based on the facts that SNP induced H_2_O_2_, iron ions, NO and O_2_^·−^ generations in both HepG2 cells (Supplementary Figure 1A, 1B, Figures 4A, 4B, 7C and 7D) and chondrocytes [23], we believed that SNP induced ^·^OH and ONOO^−^ generation in the two cell lines. However, in contrast to the dominant role of ^·^OH for the cytotoxicity of SNP in chondrocytes [23], ONOO^−^ instead of ^·^OH played a dominant role in SNP-induced apoptosis in HepG2 cells (Figure 5). It is thus reasonable to infer that HepG2 cells are very sensitive to ONOO^−^ instead of ^·^OH.

The maintenance of intracellular redox homeostasis depends on a complex web of antioxidant molecules. Tripeptide glutathione (GSH), a crucial component of cellular antioxidant defenses, protects cells against oxidative stress [41]. It was reported that GSH scavenged O_2_^·−^ and H_2_O_2_ with very slow rate constants of 10^2^~3 × 10^3^ M^−l^·s^−l^ [42–44] and 18~26 M^−l^·s^−l^ [43], respectively. However, GSH has a powerful ability to scavenge ^·^OH with a rate constant of 8.8 × 10^9^ M^−l^·s^−l^ [45]. Resistance of cell against oxidative stress is associated with high intracellular GSH levels [46–48]. It was reported that the GSH level of HepG2 cells was about ~50 ng/mg protein [49], much higher than the ~14 ng/mg protein in chondrocytes we recently measured [23]. Therefore, we here speculated that the high level of intracellular GSH in HepG2 cells could rapidly scavenge the ^·^OH from the Fenton reaction between H_2_O_2_ and iron ions released from SNP, which might be the reason why iron ions and H_2_O_2_ released from SNP were not involved in the SNP-induced apoptosis in HepG2 cells.

Peroxiredoxins (Prxs), a ubiquitous family of cysteine-dependent peroxidase enzymes, can reduce more than 90% of cellular peroxides and has been considered to be the only enzymes known to catalyze the reduction of ONOO^−^ to nitrite [50]. Moreover, Prxs were considered to be the most efficient ONOO^−^ scavengers [51]. It was reported that Prxs reacted with ONOO^−^ with constants on the order of ~10^6^×10^7^ M^−l^·s^−l^ [52–54], much higher than the reaction constant (1400 M^−l^·s^−l^) between GSH and ONOO^−^ [54]. It is well recognized that the content of Prxs is cell- and tissue-specific [55]. The fact that ONOO^−^ dominates the SNP-induced cytotoxicity (Figure 5) indicates that the endogenic Prxs of HepG2 cells are unable to effectively scavenge the SNP-induced ONOO^−^.

Our observation that iron-saturated DFO completely prevented SNP-induced cytotoxicity in HepG2 cells (Figure 7F) further confirms the notion that many physiological effects, including inhibiting ONOO^−^-mediated oxidation, of DFO are independent of metal chelation [56, 57]. Although iron ions were not involved in SNP-induced apoptosis in HepG2 cells (Figure 7E and 7F), DFO, an iron ions chelator, completely prevented SNP-induced cytotoxicity (Figure 7A). It was reported that DFO can directly scavenge ONOO^−^ and O_2_^·^− [56, 58], which was further confirmed by our findings that DFO completely scavenged O_2_^·−^ and ONOO^−^ generations (Figure 7B) in HepG2 cells. In addition, we here for the first time found that DFO could also completely inhibit SNP-induced NO (Figure 7B), strongly demonstrating the scavenging effect of DFO on NO. Moreover, we also assessed the effect of DFO on SNP-induced nitrites/nitrates variation, and found that DFO did not prevent the decomposition of SNP (data not shown). Therefore, the complete protective effect of DFO on SNP-induced apoptosis in HepG2 cells is due to the complete scavenging action of DFO on the SNP-induced NO, O_2_^·−^ and ONOO^−^ generations.

To our surprise, NAC, a widely used ROS scavenger, significantly enhanced the cytotoxicity of SNP in HepG2 cells (Figure 8E). Although NAC exhibited excellent role in inhibiting H_2_O_2_ (Figure 8B), it potently increased SNP-induced intracellular ROS/ ONOO^−^ (Figure 8B and 8D). Our results that NAC enhanced the SNP-induced an increase of the nitrites/nitrates concentration (Figure 8C) further confirmed the notion that some reducing agents such as thiols contributed the reductive biodegradation of SNP to release NO [3]. Similarly, Ottaviani and colleagues reported that NAC enhanced the cytotoxicity of SNP in fat body cells (IPLB-LdFB) [59]. Collectively, NAC promoted the decomposition of SNP to release NO and O_2_^·−^, enhancing the formation of ONOO^−^, and thus enhancing the cytotoxicity of SNP in HepG2 cells.

In reality, we also assessed the role of ONOO^−^ and ^·^OH in SNP-induced apoptosis in Hep3B cells. PTIO did not prevented SNP-induced cytotoxicity (Figure 2A) and SNPex induced the same cytotoxicity as SNP (Figure 2B), which indicated that NO was not involved in SNP-induced cytotoxicity of Hep3B cells. In contrast to the dominant role of ONOO^−^ in SNP-induced apoptosis in HepG2 cells, ^·^OH instead of ONOO^−^ dominated SNP-induced apoptosis in Hep3B cells (data not shown). We are currently focusing on exploring the roles of endogenic antioxidants such as GSH and Prxs towards SNP-induced oxidative and nitrative stress in various cell lines including Hep3B and HepG2 cell lines.

**Figure 9:**
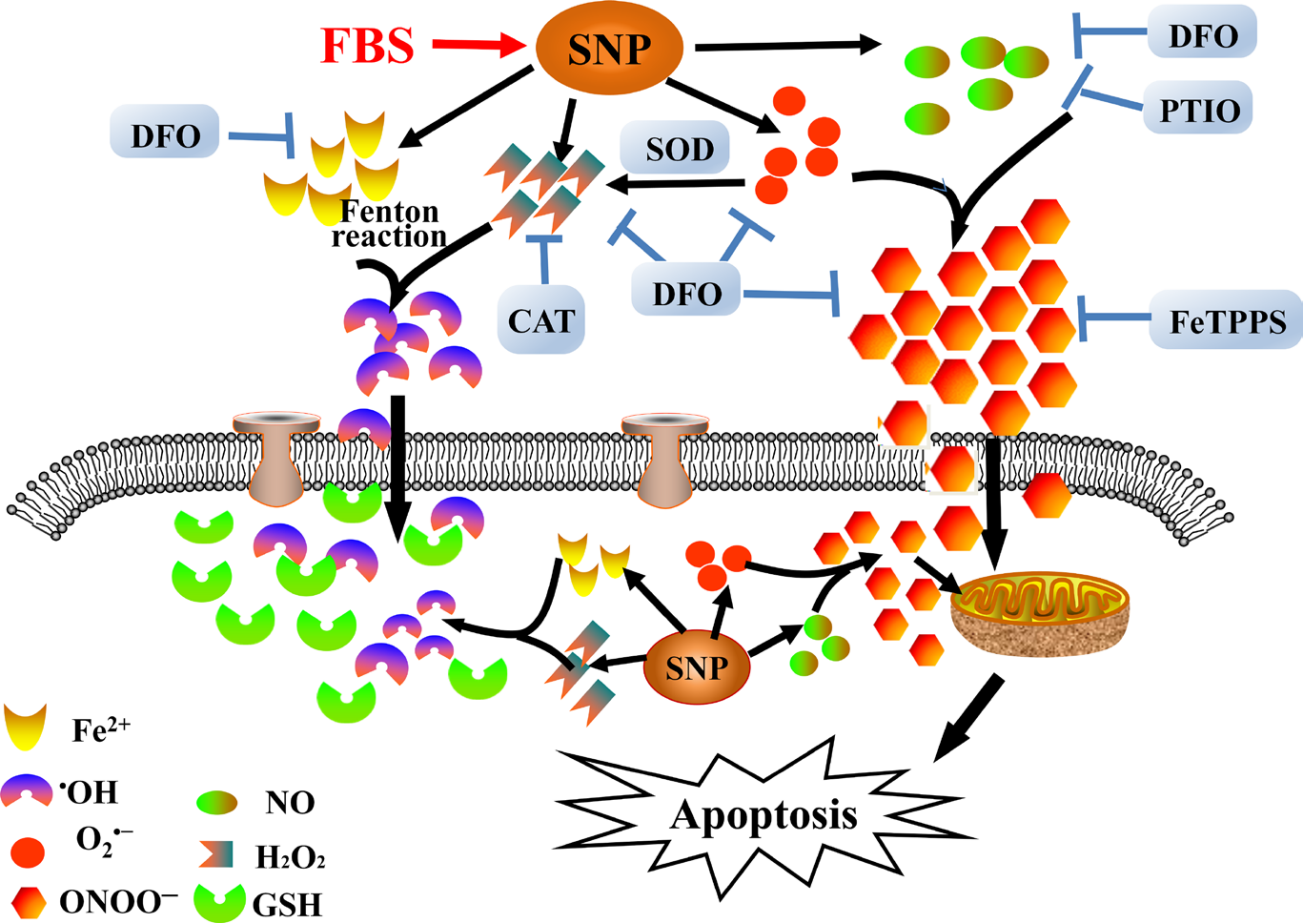
Schematic diagram showing decomposition of SNP and subsequent proapoptotic action in HepG2 cells

We summarize the metabolism products associated with the potent proapoptotic actions of SNP in HepG2 cells in Figure 9. After exposure to cell culture medium containing fetal bovine serum, SNP releases NO, O_2_^·−^, H_2_O_2_ and iron ions. Reaction of NO with O_2_^·−^ produces ONOO^−^ to dominate SNP-induced apoptosis in which both iron ions and H_2_O_2_ are not involved. In addition, DFO exhibits a very excellent inhibitory effect on SNP-induced cytotoxicity by scavenging NO, O_2_^·−^ and ONOO^−^. Sensibility of HepG2 cells to ONOO^−^ instead of H_2_O_2_ and ^·^OH may be due to the high intracellular GSH level. However, the precise biological mechanism by which ONOO^−^ dominates SNP-induced apoptosis in HepG2 cells is unclear, and we are focusing on this issue by further exploring the roles of GSH and Prxs in the response of various kinds of cell lines to oxidative stress.

## MATERIALS AND METHODS

### Materials

SNP, dimethyl sulphoxide (DMSO), Carboxy-PTIO (PTIO), N-acetyl cysteine (NAC), 2′,7′-Dichlorofluorescin diacetate (DCFH-DA), Deferoxamine mesylate salt (DFO), catalase (CAT) and Rhodamine 123 (Rho 123) were from Sigma (St. Louis, USA). Fe(III) 5,10,15,20-tetrakis(4-sulfonatophenyl) porphyrinato chloride (FeTPPS) and 2-(3,6-diamino-9H-xanthen-9-yl)-benzoic acid, methyl ester (DHR 123) were from Cayman (MI, USA). 3-(Aminopropyl)-1-hydroxy-3-isopropyl-2-oxo-1-triazene (SIN-1) and 3-morpholinosydnonimine (NOC-5) were from Enzo Life Sciences Inc (New York, USA). Dulbecco's modified Eaglemedium (DMEM) was from Gibco (Carlsbad, California, USA). Fetal bovine serum (FBS) was from Sijiqing (Hangzhou, China). 3-Amino,4-aminomethyl-2′,7′-difluorescein, diacetate (DAF-FM DA), RIPA lysis reagent, Dihydroethidium (DHE), superoxide dismutase (SOD) and Staurosporine (STS) were from Beyotime Institute Biotechnology (Jiangsu, China).

### Cell lines and cell culture

HepG2 and Hep3B cells were purchased from the Experimental Animal Center, SUN YAT-SEN University (Guangzhou, China), and were cultured in DMEM supplemented with antibiotics (100 U/ml penicillin and 100 U/ml streptomycin) at 37°C in humidified 5% CO_2_.

### Treatments

SNP powder was freshly dissolved in ultrapure water to obtain 100 mM concentration SNP solution before experiment. Then, the SNP was diluted in culture medium and added to cells to obtain various concentrations. All SNP experiments were performed in dark. Exhausted SNP (SNPex) was obtained by leaving the solution of SNP under light exposure for 24 h at room temperature as described previously [23]. NOC-5 was freshly dissolved in 0.1M NaOH. SIN-1 was freshly dissolved in PBS solution. CAT and NAC were prepared just before the experiments by dissolving the powders in ultrapure water. FeTPPS, SOD and DFO were dissolved in ultrapure water as stock solution and stored at −20°C in the dark. Iron-saturated DFO was made by dissolving equimolar amount of DFO and then ferric chloride in saline [60]. PTIO was dissolved in DMSO, and the final concentration of DMSO was less than 0.1% (v/v) in experiment. HepG2 cells were pretreated with NAC or PTIO for 2h, and CAT, SOD, DFO, FeTPPS for 30 min, and then co-treated with 1.25 mM SNP for 24 h. HepG2 cells were exposed to NOC-5 for 0.5 h, SIN-1 for 2 h. For all cell experiments, before drugs treatment, cells were replaced with fresh medium.

### Cell viability and apoptosis assay

Cell viability was detected by Cell Counting Kit-8 (CCK-8) assay (Dojindo, Kumamoto, Japan) as described previously [29]. HepG2 cells cultured in 96-well plates (1 × 10^4^ per well) for 24 h were treated with different stimuli, viable cells were assessed by absorbance measurements at 450 nm using an auto microplate reader (infinite M200, Tecan, Austria). Hep3B cells cultured in 96-well plates (1×10^4^ per well) for 24 h were treated with different stimuli, viable cells were assessed by absorbance measurements at 450 nm using the microplate reader.

Cell apoptosis was quantified by flow cytometry (FCM) (FACSCCanto II, BD Biosciences) analysis with Annexin V-FITC/PI apoptosis detection kit (Bestbio, Shanghai, China) as described previously [29]. HepG2 cells cultured in 6-well (5×10^5^ per well) plates for 24 h were treated with different stimuli, then the cells were collected and stained with 5 ml of AnnexinV-FITC and 10 ml PI for 15 min at 4°C before FCM analysis. 10,000 events were recorded for each FCM analysis.

### Measurement of mitochondrial membrane potential (ΔΨm)

Loss of ΔΨm was assessed by FCM analysis with Rho 123 staining as previously described [29]. Briefly, HepG2 cells cultured in 6-well plates for 24 h were treated with different stimuli, and were subsequently harvested and stained with 5 μM Rho 123 at 37°C for 20 min in dark, then washed with PBS twice before FCM analysis. Results were expressed as the proportion of cells with low Rho 123 fluorescence indicating the loss of ΔΨm.

### Determination of caspases activation

The CaspACE FITC-VAD-FMK In Situ Marker (Promega Corporation, WI, USA) was used to detect the activation of caspases according to the manufacture's protocol. The structure of the cell-permeable caspase inhibitor peptide VAD-FMK (Val-Ala-Asp-Phe-Met-Lys) conjugated to FITC allows delivery of the inhibitor into the cell, where it binds to activated caspase, serving as an *in situ* marker for apoptosis. HepG2 cells cultured in 6-well plates for 24 h were treated with different stimuli, and then the cells were collected and diluted in 0.5 ml PBS. The cells were stained with 1 μl of FITC-VAD-FMK (5 mM) at 37°C for 20 min in dark, and then they were washed with PBS twice before FCM analysis.

### Measurement of nitrite and nitrate

NO concentration was indirectly quantified by measuring its oxidation by-products nitrites and nitrates using the auto microplate reader just as described previously [23]. Cells cultured in 6-well plates for 24 h were treated with different stimuli, and then 50 μl cell medium of each sample was collected and mixed with 50 μl Griess reagents at room temperature for 10 min in 96-well plates. Absorbance at 540 nm was measured using the auto microplate reader.

### Measurement of intracellular ROS and NO

DCFH-DA and DAF-FM DA are cell-permeable fluorescent probes. Intracellular ROS or NO level was quantified by using FCM analysis with DCFH-DA or DAF-FM DA staining just as described previously [29]. Cells cultured in 6-well plates for 24 h were treated with different stimuli, and then cells were collected and stained with 20 μM DCFH-DA for 30 min or with 5 μM DAF-FM DA for 20 min at 37°C in dark. After washing with PBS three times, the samples were analyzed by FCM.

### Measurement of superoxide anion (O_2_^·−^) and Peroxynitrite (ONOO^−^)

DHE and DHR 123 are cell-permeable fluorescent probes. Intracellular O_2_^·−^ or ONOO^−^ level was quantified by using FCM analysis with DHE or DHR 123 staining. DHE, an O_2_^·−^ sensitive probe, reacts with O_2_^·−^ to form a diagnostic marker product (2-hydroxyethidium, 2-OH-E+). DHR 123 is oxidized by ONOO^−^ to the highly fluorescent product rhodamine. Briefly, cells cultured in 6-well plates for 24 h were treated with different stimuli, then the cells were collected and stained with 10 μM DHE for 30 min or with 10 μM DHR 123 for 20 min at 37°C in the dark. After washing with PBS three times, the samples were analyzed by FCM.

### Measurement of H_2_O_2_

H_2_O_2_ concentration was measured using the Amplite fluorimetric hydrogen peroxide assay kit (ATT Bioquest, Sunnyvale, CA) just as described previously [23]. Briefly, cells cultured in 6-well plates for 24 h were treated with different stimuli, then 50 μl cell medium was collected and incubated with 50 μl reaction mixtures provided by the kit for 30 min at room temperature. H_2_O_2_ level was detected at 570 nm in 96-well plates by using the auto microplate reader.

### Determination of iron ions

Iron ions concentration was determined using QuantiChrom™ Iron Assay Kit (BioAssay Systems, CA, USA) according to the manufacture's protocol. HepG2 cells cultured in 6-well plates were treated with different stimuli, and then cell medium and cell lysate were collected just as described previously [23]. Briefly, 50 μl of cell medium or cell lysate was mixed with 200 μl of reaction mixture provided by the kit and then incubated for 40 min at room temperature in 96-well plates. The optical density was measured at 590 nm by the auto microplate reader.

### Statistical analysis

Data were presented as mean ± SD from at least three independent experiments and analyzed using Student's *t-test*. Statistical and graphic analyses were done using the software SPSS 19.0 (SPSS, Chicago) and Origin 8.0 (OriginLab Corporation). *P* < 0.05 was defined as statistical significance.

## SUPPLEMENTARY MATERIALS FIGURES AND TABLES


