# Supplementary material for: Peroxynitrite dominates sodium nitroprusside-induced apoptosis in human hepatocellular carcinoma cells
Source: Oncotarget. 2017 Mar 13;8(18):29833–45. doi: 10.18632/oncotarget.16164 (PMC5444707; doi:10.18632/oncotarget.16164)
Supplement: Supplementary file 1 [file oncotarget-08-29833-s001.pdf]

Peroxynitrite dominates sodium nitroprusside-induced apoptosis in human hepatocellular carcinoma cells

Supplementary Materials

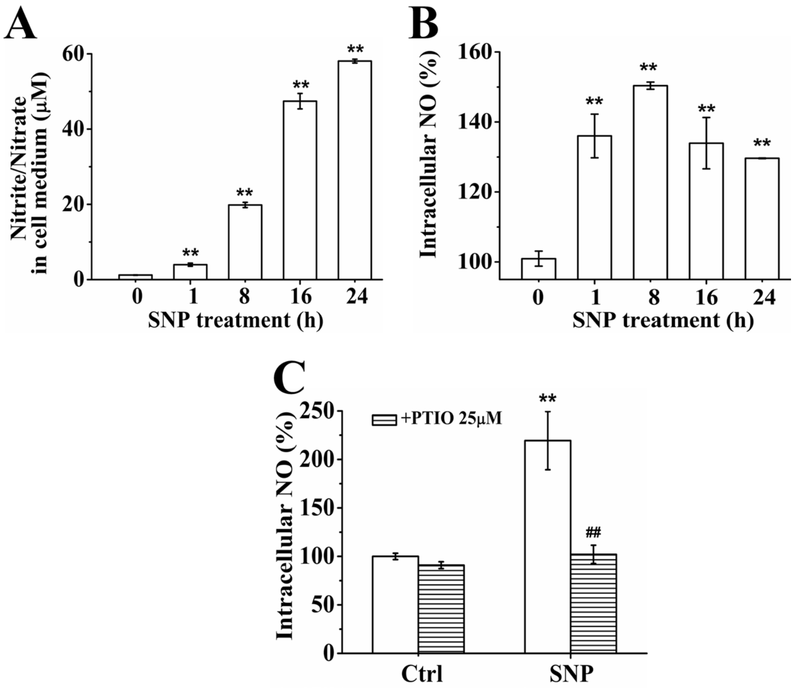

Supplementary Figure 1: PTIO inhibits SNP-induced NO generation. (**A**) SNP induced time-dependent nitrite/nitrate generation in cell medium. (**B**) SNP induced rapid intracellular NO generation. (**C**) PTIO pretreatment completely inhibited SNP-induced intracellular NO generation. Those results represent duplicates with three independent experiments. ***P* < 0.01 vs Control.

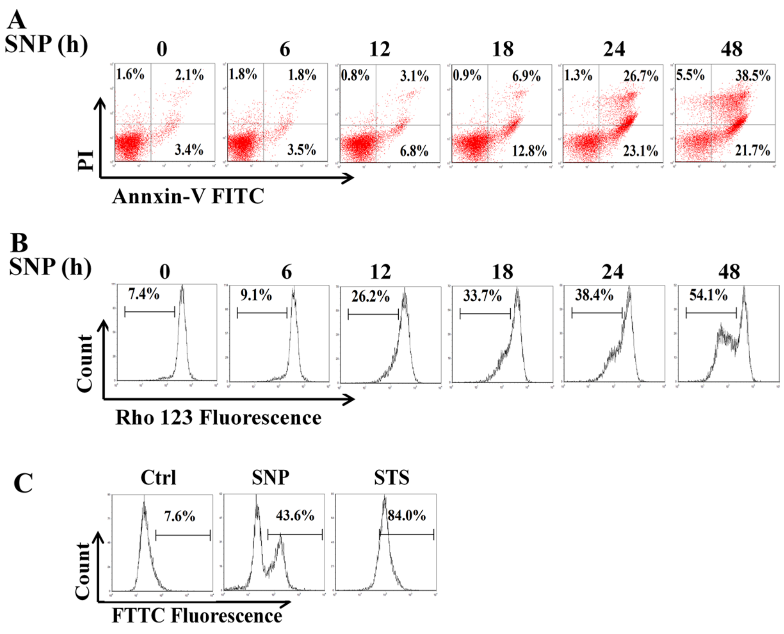

Supplementary Figure 2: SNP induces apoptosis in HepG2 cells. (**A** and **B**) SNP induced time-dependent apoptosis (A) and loss of ΔΨm (B). (**C**) SNP induced remarkable caspases activation.
